# The context of emergency contraception use among young unmarried women in Accra, Ghana: a qualitative study

**DOI:** 10.1186/s12978-018-0656-7

**Published:** 2018-12-19

**Authors:** Slawa Rokicki, Sonja Merten

**Affiliations:** 1 Geary Institute, University College Dublin, Dublin, Ireland; 2 Society, Gender and Health Unit, Department of Epidemiology and Public Health, Swiss Tropical and Public Health Institute, Socinstrasse 57, 4002 Basel, Switzerland; 3 University of Basel, Petersplatz 1, 4001 Basel, Switzerland

**Keywords:** Adolescent health, Emergency contraception, Contraception, Reproductive health

## Abstract

**Background:**

Over the past decade, awareness and use of emergency contraceptive pills (ECPs) among young women has rapidly increased in Ghana; however, the rate of unintended pregnancy among this group remains high. We conducted a qualitative study to better understand the context and patterns of ECP use among young unmarried women in Ghana.

**Methods:**

We conducted in-depth interviews with unmarried sexually active women aged 18–24 in Accra, Ghana to explore their perceptions, experiences, and opinions regarding sexual relationships and contraceptive methods, and to examine the factors that influence choice of ECPs. A total of 32 young women participated in the study.

**Results:**

Most participants had used ECPs at least once. Participants described being unable to plan for sexual encounters, and as a result preferred ECPs as a convenient post-coital method. Despite being widely and repeatedly used, women feared the disruptive effects of ECPs on the menstrual cycle and were concerned about long-term side-effects. ECPs were sometimes used as a back-up in cases of perceived failure of traditional methods like withdrawal. Misinformation about which drugs were ECPs, correct dosage, and safe usage were prevalent, and sometimes spread by pharmacists. Myths about pregnancy prevention techniques such as urinating or washing after sex were commonly believed, even among women who regularly used ECPs, and coincided with a misunderstanding about how hormonal contraception works.

**Conclusions:**

ECPs appear to be a popular contraceptive choice among young urban women in Ghana, yet misinformation about their correct usage and safety is widespread. While more research on ECP use among young people is needed, these initial results point to the need to incorporate information about ECPs into adolescent comprehensive sexuality education and youth-friendly services and programmes.

## Plain English summary

Over the past decade, awareness and use of emergency contraceptive pills (ECPs) among young women has rapidly increased in Ghana. However, the rate of unintended pregnancy among this group remains high.

In this study, we interviewed unmarried women aged 18–24 in Accra, Ghana to understand when, how, and why they use ECPs. A total of 32 women participated in the study.

Most participants had used ECPs at least once. Participants described being unable to plan for sexual encounters, and as a result preferred ECPs because they can be taken after sex. Despite being widely and repeatedly used, women feared that ECPs would cause them harm by disrupting their menstrual cycle and were also concerned about long-term side-effects. ECPs were sometimes used as a back-up in cases when women used a traditional method like withdrawal, but were worried that it had not been effective. Women were also misinformed about which drugs were ECPs, and the correct dosage and safe usage of ECPs. Pharmacists were sometimes the source of the wrong information concerning ECPs. Myths about pregnancy prevention techniques such as urinating or washing after sex were commonly believed, even among women who regularly used ECPs.

In conclusion, ECPs appear to be a popular contraceptive choice among young women in Accra, yet misinformation about their correct usage and safety is widespread. While more research on ECP use among young people is needed, these initial results point to the need to incorporate information about ECPs into adolescent comprehensive sexuality education and youth-friendly services and programmes.

## Background

Worldwide, 44% of pregnancies are unintended [1]. Unintended pregnancies increase risk of maternal and infant mortality and morbidity, as well as reduce women's prospects for education and employment [1, 2]. In sub-Saharan Africa (SSA), 30–43% of unintended pregnancies end in induced abortion, where 3 out of 4 abortions are classified as unsafe [1, 3].

Emergency contraceptive pills (ECPs) are an essential element of contraceptive services to reduce risk of unintended pregnancy. ECPs are indicated for women who have had unprotected sex, for example due to method failure, incorrect use or non-use of contraceptives, or sexual assault [4]. They have been developed and marketed as an “emergency” or “back-up” method in order to discourage regular use [5]. Over the past decade, knowledge and use of ECPs has increased in many SSA countries, particularly among unmarried women in urban areas [6]. As access to ECPs has increased, studies have found that young women may prefer emergency contraception to long-term methods of contraception, as a convenient post-coital method with fewer side effects [5, 7–9].

In Ghana, 80% of sexually active unmarried women knew about emergency contraception in 2017, up from 49% in 2008 [10, 11]. In 2017, ECPs were the most commonly used method of contraception (either modern or traditional) among sexually active unmarried women aged 20–24 [10]. Other recent studies show similar findings. A 2018 study of youth aged 15–24 in Ghana found that ECPs were the second most popular modern or traditional method among women, next to condoms [12]. A 2016 study of female Ghanaian university students found that 63% had heard of ECPs and 37% had used ECPs [13]. A 2014 study of ECP awareness and use in the northern region of Ghana found that 69% of women aged 15–49 were aware of ECPs; among these, 40% had ever used them [14].

Yet, while use of ECPs has increased among young women, sexual and reproductive health (SRH) indicators have not improved. Results from the 2017 Ghana Maternal Health Study found that nearly half of women aged 20–24 began having sex before age 18, yet less than a third of sexually active unmarried women use a modern form of contraception [10]. In this context, the rate of unintended pregnancies is high: about 16% of women aged 20–24 have ever had an induced abortion, and a third of births to women aged 20–24 are mistimed or not wanted at all [10, 15]. About 14% of women aged 15–19 have begun childbearing, a proportion that has remained stagnant since 1998 [10].

Given the growing awareness and use of ECPs in Ghana and other countries in SSA, it is important to examine why increased use of ECPs has not translated into improved SRH among young women. The aim of this qualitative study is to better understand the context and patterns of ECP use among young unmarried women in Accra, Ghana. We explore women’s perceptions, experiences, and opinions regarding sexual relationships and contraceptive methods, and examine the factors that influence choice of ECPs. Finally, we discuss our results in the context of expanding sexuality education curricula as well as national reproductive health policy guidelines to incorporate information about ECPs.

## Methods

### Study setting and participants

This study was conducted in April 2016 in Accra, Ghana. The participants were drawn from a sample of participants who completed a randomized controlled trial in Ghana that investigated the effectiveness of sexuality education text messaging programs on knowledge of SRH and on sexual behaviour [16, 17]. Briefly, the original study sent informational text messages about reproductive health to participants for 12 weeks. The inclusion criteria for the original trial was girls aged 14–24 in their second year of secondary school in the Greater Accra region. Participants completed self-administered questionnaires at baseline, midline (3 months later), and endline (15 months later).

The study found that the text messaging programs improved knowledge of reproductive health, but did not have a clear and significant impact on sexual behaviour or use of contraception, compared to the control group. In order to further understand the results of the study, we conducted in-depth interviews one year after the end of the original trial. We used purposive sampling, inviting women who were in the treatment arms of the original trial (i.e. had received sexuality education text messages) and who had reported that they had sex in the past year at endline. We also sampled those who claimed they had ever been pregnant at endline in order to understand the contexts of those pregnancies.

### Study design

We conducted in-depth individual interviews following a topic guide. Topics included perceptions about community norms surrounding sex and contraception, personal sexual experiences, attitudes about and experiences with use of modern and traditional methods of contraception, perceptions of barriers to use of contraception, experiences with pregnancy and abortion, experiences and community norms surrounding ‘sexting’ (exchanging explicit sexual messages via mobile phones), and feedback on the text messaging program they were involved in. The average interview length was 53 min with a range of 32 min to 1 h 34 min.

Interviews were conducted in local language (Twi, Ga, or Ewe) or English based on respondent preference. Two female interviewers of about the same age as respondents were trained on qualitative methods of interviewing in a 2-day training. Training materials were adapted from a field guide created by Family Health International [18]. Interviews were audiotaped, and then translated into English and transcribed simultaneously by a professional Ghanaian translator. To ensure validity, the senior field manager verified a sample of the translations.

We obtained written informed consent from each participant. Participants were informed that participation was voluntary, they could stop the interview at any time, and data would be treated confidentially. The recorded interviews did not include the participant’s name. As a token of appreciation, every respondent received 5GHC in airtime phone credit (about 1.30USD). Participants were also provided a free hotline phone number, operated by Marie Stopes International, in case of any further questions or concerns about SRH or their health. Institutional Review Board (IRB) approval was granted by the Committee on the Use of Human Subjects in Research at Harvard University (IRB13–1647) as well as the Ghana Health Service Ethical Review Committee (GHS-ERC:05/09/13). Thirty-two women signed a letter of consent and the final sample included all 32.

### Data analysis

The interviews were analyzed using a thematic approach that allowed for themes to emerge through an iterative process of coding and discussion [19]. A preliminary list of codes was created using the topic guide. Transcripts were then independently reviewed by two members of the research team who assigned preliminary codes. The research team members met and discussed their findings, and codes were subsequently added, removed, and refined during these discussions. This process continued until the research team agreed on the final themes and categories. To ensure reliability, all cases of conflicting results were discussed thoroughly in an open process until consensus was reached. This process included comprehensive data treatment and deviant case analysis, in which research team members actively sought out and identified similarities and differences across accounts to ensure different perspectives were represented [20].

Data management and analysis were conducted using the computer software package NVivo™ software Version 11 (QSR International, Doncaster, Victoria, Australia). The study preserved a well-documented audit trail of materials to ensure transparency.

## Results

The demographic characteristics of the participants are shown in Table 1. Participants were between 18 and 24 years of age. All 32 participants were unmarried; however 97% were in a relationship. The median age at first sex was 18 years. Six participants had gotten pregnant since becoming sexually active; all six had an induced abortion. None of the participants had children.

**Table 1:** Demographic characteristics of women participating in in-depth interviews

Characteristic	Total *N* = 32
Age, years [median (range)]	20 (18–24)
Religion [n (%)]	
Muslim	6 (19)
Catholic	2 (6)
Protestant	8 (25)
Pentecostal/Charismatic	16 (50)
Ethnicity [n (%)]	
Akan	10 (31)
Ga	7 (22)
Ewe	7 (22)
Other/Missing	8 (25)
Mother’s education [n (%)]	
Less than secondary	17 (53)
More than secondary	10 (31)
Don’t know/Missing	5 (16)
Father’s education [n (%)]	
Less than secondary	4 (13)
At least secondary	18 (56)
Don’t know/Missing	10 (31)
Marital status [n (%)]	
Married	0
Currently in a relationship	31 (97)
Single	1 (3)
Age at first sex [median (range)]	18 (15–20)
Ever pregnant [n (%)]	6 (19)

*Notes*: Information on age, marital status, age at first sex, and ever pregnant was obtained from in-depth interviews. Information on religion, ethnicity, and parental education was obtained from baseline data from the original trial from which respondents were recruited [16]

The most common forms of contraception that participants had tried at least once were condoms and ECPs, followed by withdrawal and using a menstrual calendar. Only 5 participants mentioned using oral contraceptive pills (OCPs) and only 1 had used an injectable. The most common ECP brand was Postinor-2® (Levonorgestrel); others that were mentioned were Lydia Postpil® (Levonorgestrel), Lenor® (Levonorgestrel), and (incorrectly) Primolut-N® (Noresthiesterone). Primolut-N is misused off-label as an ECP or a pre-coital contraceptive in Ghana [4, 21, 22].

Themes arising from the interviews are shown in Table 2. The major themes that emerged were “unwanted and unplanned context of sexual encounters”, “popularity of ECPs”, “ECPs as back-up for traditional methods”, “negative perceptions of side effects of ECPs”, “misinformation about ECPs”, “negative perceptions of condoms”, and “misunderstanding of human reproduction and contraception”.

**Table 2:** Themes arising from in-depth interviews

Theme	Categories
Unwanted and unplanned context of sexual encounters	Coerced or pressured sex
	Sex in return for financial benefits
	Impromptu sex
Popularity of ECPs	Post-coital method
	Diffusion of information about it
	Can take without partner's knowledge
	ECPs easier to take than OCPs because fewer pills
ECPs as back-up for traditional methods	Lack of understanding of calendar method
	Lack of trust in partners to practice withdrawal correctly
	ECP can be used as backup in case of failure
Negative perception of side effects of ECPs	Dislike/fear of change in menses
	Fear/lack of clarity of side effects
	Fear of “addiction” / pills will become ineffective
Misinformation about ECPs	Lack of clarity as to which drugs are ECPs vs OCPs
	Misunderstanding about how to take ECPs
	Pharmacist providing incorrect information
	Difficulty in obtaining ECPs due to cost, shyness, or pharmacist refusal to sell
Negative perception of condoms	Negative representation in society (representing promiscuity or infidelity)
	Lack of trust in condoms
	Reduced sexual pleasure
Misunderstanding of human reproduction and contraception	Pregnancy prevention myths
	Lack of understanding about how contraceptives work
	Lack of timely sexual health education or parental support/communication

*Notes*: *OCP* Oral contraceptive pill, *ECP* Emergency contraceptive pill

### Unwanted and unplanned context of sexual encounters

Participants described sexual encounters as largely unplanned for a variety of reasons. Coerced or pressured sexual encounters were mentioned by the majority of participants, either as something they experienced themselves or something they noticed in the community. While six participants described having been physically forced into sexual activity, most described these encounters as partners putting pressure on them to have sex over time. Participants described their first time having sex as necessary to prove their love to their partner or else risk dissolution of the relationship.

“Well, to me, he was like, we should try it. And I said, no, I wasn’t ready for that. He was like, “for the first time then…” – as for him he thinks I don’t love him because we’ve dated for 2 years without sex.” (Age 21)

Many participants also described sex as an obligation in return for financial benefits. Participants described feeling indebted to boyfriends who provide schooling fees or take care of them financially, and feeling that eventually they had to repay the boyfriend with sex. One participant said,

“Because of the hardship I was going through that was why I went in for a guy, so if he provides my basic needs then if he ask me for sex and maybe I don’t give him, I will think he will go for another girl, which means I will forgo my basic needs.” (Age 22)

Another participant described a common situation in her neighbourhood:

“Girls may need something like pads, when you ask your mother and she doesn’t get it for you, you will definitely go and ask a guy. He will give it to you but not for free. It’s only a few that will do that for free. When he gives you today, tomorrow he will demand he have sex with you first.” (Age 18)

Finally, participants described sexual encounters, even wanted, as unexpected. Unplanned encounters happened with both regular partners and via casual sex. For example, a participant described her inability to plan for sex:

“I think sorry to say, you don’t prepare to have sex, that’s why I did it. We don’t prepare to have sex. You can say you’re not going to have sex with your man or your woman or your wife; but it is feelings…Yes, you can just have sex without knowing. You know you are doing something but you can’t control it, because it’s sex. Yes. So, that’s what happened.” (Age 20)

Previous research has found that when sexual encounters are unplanned, particularly with an imbalanced gender dynamic, young women are less able to control their sexual experiences including the use of condoms and other preventative forms of contraception [23, 24].

### Popularity of ECPs

Twenty-six of the 32 participants had used ECPs at least once. The most common brand was Postinor-2. Participants felt that ECPs were easy to take since they could be taken after having sex, rather than planning for it. Others said that aside from condoms, Postinor-2 was the only type of contraception (outside of condoms) they knew about. A number of participants mentioned hearing recommendations about ECPs from friends or aunties. Upon being asked why a participant chose to use Postinor-2, she replied,

“That was the only one I know of… I asked my friend and she told me that one was very good so I should try it. So when I tried it I realized it was good.” (Age 20)

Additionally, some participants preferred ECPs so that they could take it without their partner’s knowledge. Some participants described how a partner wanted them to become pregnant, even though they were not ready, so they took ECPs without informing their partners. One participant described her situation:

“For him he said he wants a child but I told him I’m not ready yet so if I tell him to do [use a condom], he wouldn’t do it but on my side, I prevent myself [from getting pregnant].” (Age 22)

Finally, some participants chose to use ECPs over OCPs because they felt they could not trust themselves to remember to take a pill each day. One participant said,

“I chose Postinor-2 because the other ones I know; something like Secure [a brand of OCP] they said you will take it every day. And when you missed one day you can get pregnant. So I prefer Postinor-2 after having sex within 72 hours you have to take it. So after me having sex then I take it and then I would be free. I prefer that than Secure because I may not get the time to take the medicine everyday.” (Age 19)

### ECPs as back-up for traditional methods

While the majority of participants had attempted traditional calendar or withdrawal methods, they also described being fearful of the inefficacy of these methods. Many did not trust men to practice withdrawal correctly, and were afraid of becoming pregnant after its use. Some participants discussed taking ECPs after a perceived failure of withdrawal; for example, one 20-year-old participant had the following exchange with the interviewer:

Respondent: I think when the guy is about to release, the lady realizes but the guy because of the fantasies it doesn’t occur to him then he will release inside and pretend as if he didn’t.

Interviewer: So using this method that you are saying you have used before were you scared of getting pregnant?

Respondent: I was really scared.

Interviewer: So what did you do?

Respondent: It was after that I went to buy Postinor-2 and took it just to prevent anything.

Similarly, for the calendar method, many participants described not understanding how to calculate their safe period, particularly when their menstrual cycle changes. At the same time, participants were eager to understand these methods and felt that they had not been provided any information on them.

### Negative perceptions of side effects of ECPs

Fears, dislikes, and misunderstanding of hormonal methods (both OCPs and ECPs) were mentioned by most participants. Participants mentioned both a dislike and a fear of any change in the schedule or heaviness of flow of their menses. Participants admitted that they were not sure about side effects of hormonal methods, and during the course of the interview, a few asked about the side effects of ECPs in particular. A number of participants mentioned fear of hormonal pills, including ECPs, as damaging the womb and preventing future ability to conceive. One participant explained,

“Definitely, everything has its disadvantage. I have learnt that taking too much of Postinor-2, it will come to a time you can’t give birth.” (Age 18)

Other participants believed that taking too many pills would lead the participant to become “addicted” to them in such a way as for the contraception to become ineffective at preventing pregnancy. One participant discussed why she stopped taking Postinor-2:

“Because if you continually take the tablets or the pills, it will get to a point where your body becomes used to; so it would not work… And also I know that it will get to a point where I will get complications with it because in everything there is a bad side and there is a good side. So I can say I’m using [Postinor-2] to prevent pregnancy but maybe I may develop some infections from it. So I’ve stopped using it.” (Age 19)

While both ECPs and OCPs elicited similar reactions, ECPs appeared to be more tolerated, most likely due to the low number of pills (usually two) and therefore the relatively short-term effect they had on the menstrual cycle as compared to OCPs. Participants did not mention dosage at all; instead taking “too much” medication referred to taking too many individual pills. One respondent mentioned that she had thought about taking OCPs but was concerned about how taking a pill every day would impact her health. The idea that taking a medicine occasionally rather than daily would imply that the health impact is less has been found in other studies of ECPs in West Africa [25].

### Misinformation about ECPs

Misinformation about how to safely use ECPs was prevalent among participants, some of which was spread by pharmacists. For example, one participant described why she stopped using Postinor-2:

“Where I’ve been buying [Postinor-2], the pharmacist advised me that Postinor-2 must be taken [just] once in a year. That’s what he told me.” (Age 22)

Some participants were confused as to how to take ECPs, for example, they described taking both pills of Postinor-2 at the same time (whereas the correct dosage is one pill as soon as possible after unprotected sex, and the second pill 12 hours later). Pharmacists also misinformed participants about how to correctly take ECPs. There was also confusion among participants as to which drugs were ECPs. As previously mentioned, Primolut-N, which is indicated for treatment of endometriosis, menorrhagia and dysmenorrhea, is used off-label both as a pre-coital method and an ECP in Ghana, though the effectiveness has not been studied and no health organization or agency has recommended its use [4, 21, 22].

About half of participants mentioned difficulty in buying ECPs at drug shops due to their own shyness, the cost, or the pharmacist’s refusal to sell it, while the rest thought it was easy to acquire.

### Negative perceptions of condoms

Participants had largely negative perceptions and experiences with condoms, a finding consistent with previous qualitative literature [26]. First, many participants felt that condoms represent distrust and lack of love and commitment in a relationship. One participant said about asking a partner to use a condom:

“Some would say maybe if you use – maybe if you tell the man, he will say you are not that into me that is why you want us to use the condom, that’s what I really know. Somebody will say you don’t love me that’s why you are saying or maybe you think I have a sickness that’s why you want us to use a condom.” (Age 22)

Use of condoms also represented promiscuity on the part of women; participants said that if a male partner wanted to use a condom, it was because he thought the woman was cheating or, in one participant’s opinion, that he thought of the woman as a prostitute.

Additionally, a number of participants mentioned a lack of trust in the effectiveness of condoms. Participants feared that condoms may easily tear and burst. Some participants were fearful of the condom tearing without their knowledge and resulting in unintended pregnancy; one participant mentioned that a condom bursting would be harmful to the womb.

Finally, 23 of the 32 participants mentioned that condoms reduce the pleasure of sex, either as their own opinion, their partner's opinion, or as community norms. Many participants mentioned the analogy of using condoms to “eating toffee in a wrapper”. Similar findings were found among studies with youth in Uganda [27], South Africa [28], and Nigeria [29], and among married women in Malawi [30].

### Misunderstanding of human reproduction and contraception

Participants in general had a considerable misunderstanding of the biological process of how women become pregnant, leading to a wide variety of pregnancy prevention myths. Common myths included drinking a large amount of water or a sugary beverage to “urinate out” sperm, standing up quickly after sex, using a finger to pull out the sperm, and taking paracetamol. Similar myths have been documented in previous studies in both Ghana and other countries [5, 31, 32]. The myths also coincided with misunderstandings about how hormonal contraception works. For example, one 18-year-old participant described using ECPs regularly in order to prevent pregnancy. When asked how ECPs worked, she said,

“After sex when you take those ones it washes away the sperms from your system. It comes in a form of urine.”

Then she went on to describe preventing pregnancy when she does not use contraception:

“But sometimes, it’s when I don’t take the [EC] pill. I drink a lot of water after sex. … It also helps me urinate [the sperm out]”

Other participants described similar misunderstandings regarding how ECPs work, and in general, the way that hormonal contraceptives work to prevent a pregnancy was not known to most participants. Most participants felt that they had not received enough education about SRH. At the time they began having sex, many participants said they were in Junior High School rather than Senior High School, and that therefore their education on sexual health was inadequate. While average age at first sex is 18 years for girls in Ghana, it is common for girls around this age to be attending Junior High School due to late entry to school, repetition, and drop out and drop in [33].

## Discussion

The results of this study provide evidence on how the context of sexual encounters and the perceptions and experiences of contraception among young sexually active unmarried women in Accra, Ghana influence the use of ECPs. A number of important conclusions emerged. First, sexual encounters are often unwanted or unplanned. Young women rely on their partners financially for school fees and personal items, providing sex in return. This may reduce women’s control over the use of condoms and preventative methods of birth control, and drive women towards the use of ECPs which are within their sphere of control. A review of 45 quantitative and qualitative studies in SSA found that adolescent girls engage in sexual relationships with older men for financial benefits; while girls have, to a great extent, control over choice of partners, duration of relationships, and the start of sexual relations, they have little control over sexual practices within partnerships including condom use and violence [24]. Second, despite the widespread use of ECPs among participants, ECPs are nevertheless mistrusted due to the disruptive effect they have on the menstrual cycle. There is a pervasive belief that taking too many hormonal pills will have a negative effect on future fertility; there is also a belief that ECPs will become ineffective at preventing pregnancy if taken too many times. These findings are consistent with other qualitative studies [7, 34, 35]. Third, traditional methods such as withdrawal and following a menstrual calendar are used; however, when there is a perception of method failure, ECPs are sometimes used as a back-up method. Finally, there is a great deal of misinformation about ECPs, including which drugs are ECPs, how to take ECPs, and how frequently ECPs can be safely taken, some of which was spread by pharmacists.

We found that a large majority of participants had experience using ECPs; participants liked that ECPs could be used post-coitally and without their partner's knowledge. While the rate of use may be higher in our study because participants were sent an informational message about ECPs during the course of the original trial, many participants mentioned hearing about ECPs from friends and family. We also found that there is a substantial misunderstanding of the biology of human reproduction, which leads to myths about nonsensical pregnancy prevention techniques such as urinating out sperm. These myths are widespread and are believed even among women who regularly use modern contraception. A study of Nigerian University students also found that women combine ECPs with saline solution douches or with other medications to increase their certainty of preventing a pregnancy [34, 35]. Interestingly, participants in our study had received information on the ineffectiveness of some prevention techniques, including bathing after sex and standing up during sex, during the original trial [16, 17]. While the messaging intervention was effective at improving overall SRH knowledge, it appears that these messages were not comprehensive enough to dispel the widespread myths concerning pregnancy prevention.

Our study contributes to the larger literature on ECPs in SSA. Recent studies have found that levels of awareness and use of ECPs among young women vary considerably across countries in SSA [8, 36–39]. Consistent with the findings of this study, previous research has found that young women generally find ECPs an acceptable and convenient option that fill the need of a post-coital contraception that can be taken as needed [4, 5, 7, 25]. However, our results also contribute to the growing evidence on barriers to ECP access and use, including myths and misconceptions about risks of ECPs [5, 25, 34, 35, 40], poor knowledge of correct usage [13, 34, 39], and restrictive distribution practices of pharmacists and health care providers [41–44].

A critical step forward to reducing misinformation and misuse of ECPs would be to incorporate information on ECPs into comprehensive sexuality education (CSE) in schools. Ghana adopted an Adolescent Reproductive Health Policy in 2000, which led to the inclusion of a reproductive health component in the educational curriculum at primary, junior high, and senior high levels; however, a 2017 report on implementation of SRH education policies in Ghana found that fewer than half of the students reported that they had learned about contraceptive methods [45]. The majority of students who started learning SRH in senior high school said that they would have liked to have started earlier, a finding that is supported in our study. Among teachers, only 54% reported teaching about ECPs at all, while 99% reported teaching about condoms and 83% reported teaching about OCPs [45]. Educational curricula should include a wider range of contraceptive options, along with information on the mechanisms of how methods work and a comprehensive, accurate discussion of their side effects. In addition, our findings provide more evidence that gender and power need to be addressed within CSE, including topics of partner communication, equitable relationships, and harmful gender norms [46, 47]. Finally, as young women demonstrate high rates of pharmacy utilization for obtaining contraceptives [48], pharmacists and health care providers also need comprehensive training on correct dosage, safe usage, and side effects of ECPs. Previous research has found low knowledge of ECPs among providers in Ghana [41] and Senegal [49].

Lastly, reproductive health policies need to address the growing access to and use of ECPs among youth. The Ghana Health Service Family Health Division 2015 Annual Report, for example, details a Family Planning Programme describing trends and progress in uptake of long-acting contraceptive methods, OCPs, injectables, male and female condoms, and permanent methods, but makes no mention of ECPs at all [50]. Moreover, guidelines that address ECPs should take the social and sexual context of young women’s sexual experiences as well as their preferences into account. While ECPs were originally developed as an “emergency” method, our results support the findings of other studies that young women prefer ECPs as a post-coital method and use it repeatedly [5, 7, 8]. While ECPs are not as effective as preventative methods of contraception, repeated use has been found to be safe, though more evidence is needed [51]. Results from our study show that women are reluctant to use condoms and OCPs consistently, so the idea of “bridging” young women who use ECPs to long-term preventative methods of contraception may be challenging, a finding consistent with previous literature [4, 5]. We also find that young women are fearful of overuse of ECPs and are worried about their side effects. Therefore, misinformation about the dangers of repeated use and side effects of ECPs may lead women to rely on ineffective and possibly dangerous “prevention” methods (like urinating or taking a large dose of paracetamol). Conversely, encouraging young women to use ECPs regularly may increase the spread of sexually transmitted infections (STIs). Overall, more research is needed to understand how uptake of ECPs affects rates of STIs.

### Strengths and limitations

Our study has strengths and limitations. First, as this is a qualitative study, our results are not generalizable to other contexts and populations beyond young unmarried women in Accra, Ghana. In addition, participants in our study were originally recruited as secondary school students in Accra, thus our results also may not generalize to other demographic or geographic groups in Ghana. However, we find that characteristics of our sample, such as median age at first sex, are similar to those of women aged 20–24 in Ghana [15]. In addition, many of our findings are consistent with those of other studies conducted in Ghana [5, 7, 13]. Second, participants received a 12-week sexuality education intervention as part of the original trial, which may have impacted on their behaviour and thus their sexual experiences and preferences. However, this is a strength of our study as well in that interviewer debrief notes indicated that the fact that participants had received information from the sexuality education program (with which the interviewer was affiliated) allowed participants to feel more comfortable with sharing personal sexual information with the interviewer. For example, six participants provided long and detailed stories about their abortion experiences, indicating that they trusted the interviewers to discuss this information. Since sex is often a taboo topic in Ghana, interviewer trust is essential to obtaining complete and honest responses and ensuring credibility of the study.

## Conclusions

Continued investment in adolescent CSE, youth-friendly health services, and effective youth and community programs are essential to protect sexual and reproductive health and rights [52–54]. However, for programs  to be effective they must be tailored to the particular needs of sexually active youth. Our results show that ECPs are an important component of a comprehensive SRH strategy that is currently left out of policy and educational guidelines. Young women’s preference for ECPs may be at odds with HIV prevention approaches that solely promote condoms. The development of a youth SRH strategy must consider the social and sexual context of ECP use among young women, as well as how best to integrate ECPs into a dual-protection strategy to reduce risk of unintended pregnancy as well as HIV/AIDS and STIs.

## Abbreviations

CSE: Comprehensive sexuality education
ECP: Emergency contraceptive pill
IRB: Institutional Review Board
OCP: Oral contraceptive pill
SRH: Sexual and reproductive health
SSA: Sub-Saharan Africa
STI: Sexually transmitted infection
